# Treated Choroidal Melanoma with Late Metastases to the Contralateral Orbit

**DOI:** 10.4137/cpath.s767

**Published:** 2009-04-03

**Authors:** Sonia George, Carole A. Cooke, Gerald F. Mc Ginnity, Steve White, Laksmi Venkatraman

**Affiliations:** 1 Department of Ophthalmology; 2 Department of Histopathology, Royal Victoria Hospital, Grosvenor Road, Belfast, U.K. BT12 6BA

**Keywords:** choroidal melanoma, metastases, orbit, proptosis

## Abstract

Choroidal melanoma is the commonest adult primary intraocular tumour,^1^ and usual sites of secondary spread are to liver, bone and lung. Although delayed recurrence of ipsilateral orbital melanoma is well documented, metastasis to the contralateral orbit is a rarely encountered phenomenon. We describe a case of metastatic spread to the contralateral orbit in a patient 12 years after proton beam radiotherapy of choroidal melanoma.

## Case Report

A 73 year old lady attended ophthalmic outpatients in 1993 describing photopsia in her left pseudophakic eye. Snellen visual acuity was right eye 6/9, left eye 6/18. Dilated fundoscopy demonstrated an elevated, pigmented mass in the superotemporal quadrant of her left eye approximately 3 disc diameters from the optic nerve head. The clinical appearance was consistent with malignant melanoma of the choroid and ultrasound (U.S.) B scan demonstrated a lesion of 14 mm width and 7 mm height. Haematological markers, chest radiography and liver ultrasound were all normal. Following referral to a national centre for ocular oncology, a course of proton beam radiotherapy was instituted and the patient attended for regular follow up. Tumour dimensions were repeatedly assessed and found to be stable and regular clinical review until June 2005 demonstrated no change in the fundal mass, or anterior extension in relation to scleral markers inserted at the time of proton beam radiotherapy. No scleral extension was evidenced on thorough orbital ultrasound, nor was there any evidence of systemic involvement.

**Figure 1.**
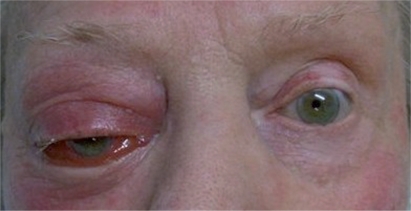
Right eye: non-axial proptosis of globe.

**Figure 2.**
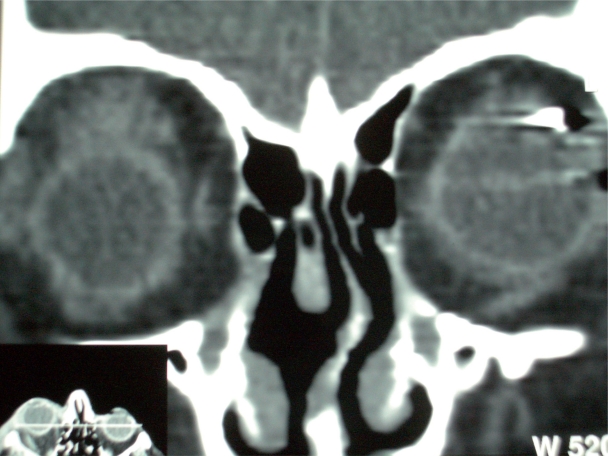
CT Scan Orbits: mass in right orbit.

**Figure 3.**
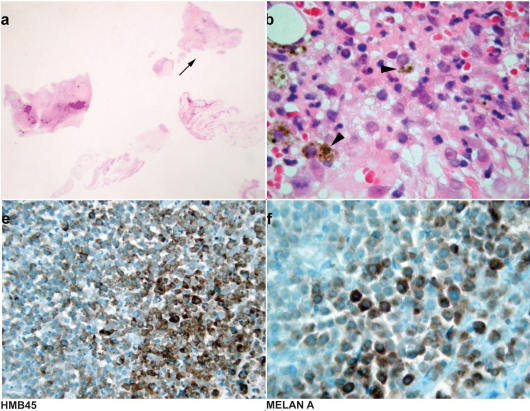
**A**) Section of the orbital biopsy showing fragments of necrotic tumour. **B**) Pleomorphic tumour cells with prominent nucleoli. Some tumour cells contain melanin pigment. **E**) HMB45 stains the viable tumour cells while the necrotic areas are unstained. **F**) MELAN A stain within viable tumour cells.

In July 2005, the patient now aged 85, presented with sudden, non-axial, painless proptosis of the right globe associated with upper lid ptosis. There was 2 mm proptosis and medial and inferior displacement of 2 mm and 8 mm respectively (Fig. 1). There was associated oedema and erythema of the soft tissue but no significant pain or heat on palpation. Orbital CT displayed a well-defined, non-enhancing, rounded mass lesion in the superolateral right orbit extending medially and inferiorly into the intraconal space displacing the superior rectus and optic nerve (Fig. 2). Histology of the biopsied lesion showed a necrotic malignant melanoma composed of epithelioid cells containing large nuclei with prominent nucleoli and a moderate amount of pink cytoplasm. Focally the cells contained melanin pigment. These expressed the melanocytic markers melan A and HMB 45 on immunohistochemistry but were non-reactive to S100 and CAM 5.2. (Fig. 3).

Systemic evaluation with CT scan of chest, abdomen and pelvis showed multiple metastatic deposits in the liver and at least one in either lung base. Referral to the regional oncology unit ensued and palliative chemotherapy of oral Temozolomide was commenced. Additional treatment with oral dexamethasone 4 mg daily greatly improved the right orbital tissue swelling and globe proptosis and the patient remained comfortable for 6 months. She died one year following this presentation.

## Discussion

Orbital metastases most commonly originate from lung, breast, kidney and prostate malignancy, and account for around 2%–9% of orbital neoplasms.^1^ They occur most frequently in females in the 5th–7th decade. Of melanotic lesions, cutaneous growths account for 5.3%–10.5% of all orbital metastases, versus 1.8%–2.9% for uveal primaries.^3^ Choroidal melanoma metastasising to the contralateral orbit is rare.^2^–^5^,^7^–^9^

Studies report a mean age range of patients with choroidal melanoma metastatic to the orbit of 54.2–61 years old.^5^,^6^ The time interval between diagnosis of the primary lesion and subsequent detection of orbital metastases for the majority falls between 6 months–10 years,^4^ with an average of 3 years.^6^ At 12 years, this patient shows one of the longest intervals between primary diagnosis and subsequent contralateral orbital spread. Four reported cases are longer; ranging from 15 to 40 years.^4^,^7^,^8^,^9^ Choroidal melanoma is undoubtedly aggressive, an estimated 70% of patients developing metastatic disease within 5 years of treatment of the primary.^2^

Papers often describe overt systemic spread preceding orbital metastases from choroidal melanoma.^3^,^6^ Unusually in this patient, an orbital secondary lesion was the first sign of remote spread. Until 1994, only 5 cases had been described demonstrating this pattern of secondary spread.^8^ Regardless of how the disease evolves, the appearance of metastases heralds imminent demise, mean patient survival being less than one year.^6^ The propensity for orbital metastases also appears unrelated to the form of treatment employed for the primary, literature review shows roughly equal numbers of patients having undergone enucleation compared to other modalities.

Although this patient was pseudophakic when diagnosed, preoperatively, a thorough fundal examination had been performed and was satisfactory. Related literature is restricted to cataract extraction after melanoma treatment. One case series reported an incidence of 8.3% tumour metastases following cataract extraction in eyes with treated choroidal melanoma.^10^ In the full cohort of 72 patients, the mean time from melanoma treatment to cataract surgery was 21.5 months. The Collaborative Ocular Melanoma Study Group found that cataract surgery in eyes treated with iodine 125 brachytherapy for choroidal melanoma did not adversely affect mortality or metastases.^11^ Here, the interval from treatment to cataract surgery was undetermined, but for patients having cataract extraction earlier than 5 years post melanoma treatment, the time span from the report of visually significant lens opacity to surgery was 1.5 years.^11^ We therefore would not recommend cataract surgery in an eye with a melanoma unless it has been shown to be successfully treated and stable for at least one year.

Although rare, contralateral orbital spread of choroidal melanoma is becoming an increasingly recognised clinical phenomenon. In contrast, post-mortem studies estimate that 1/3 of patients dying from disseminated melanoma (cutaneous and choroidal) have eye and orbital metastases, possibly due to terminal disease spread^3^. In view of the rarity of choroidal melanoma spreading to the contralateral orbit, and the underlying systemic spread often concurrent with ocular and orbital metastases, we do not recommend regular screening with costly MRI scans. Standard screening for liver and lung metastases are at present more appropriate. Earlier detection of metastases may prolong patient survival but all previous reports suggest a uniformly poor prognosis for this aggressive condition. This case demonstrates how systemic spread may manifest many years following primary presentation, and can evade continued rigorous follow up, reinforcing the need for careful patient counselling regarding prognosis of this unpredictable and insidious disease.

## References

[1] Shakin EP Shields JA The Eye and Ocular Adnexa in Systemic Malignancy. Cancer Metastatic to the Orbit Duane’s Clinical Ophthalmology 1989 5 34 4

[2] Gupta M Rennie IG Correspondence Orbital metastasis from a choroidal melanoma Eye 2005 19 227 229 15218521 10.1038/sj.eye.6701445

[3] Zografos L Ducrey N Beati D Schalenbourg A Spahn B Balmer A Metastatic Melanoma in the Eye and Orbit Ophthalmology 2003 110 11 2245 2255 14597536 10.1016/j.ophtha.2003.05.004

[4] Coupland SE Sidiki S Clark BJ McClaren K Kyle P Lee WR Metastatic Choroidal Melanoma to the Contralateral Orbit 40 Years After Enucleation Arch Ophthalmol 1996 114 751 756 8639093 10.1001/archopht.1996.01100130743022

[5] Drummond S Fenton S Pantilidis EP Harnett AN Kemp EG Correspondence. A case of cutaneous melanoma metastatic to the right eye and left orbit Eye 2003 17 420 422 12724707 10.1038/sj.eye.6700360

[6] Orcutt JC Char DH Melanoma Metastatic to the Orbit Ophthalmology 1988 95 8 1033 1037 3231441 10.1016/s0161-6420(88)33061-7

[7] Singh AD Shields JA Shields CL Takami Sato Choroidal Melanoma Metastatic to the Contralateral Choroid Am J Ophthalmol 2001 132 6 941 943 11730671 10.1016/s0002-9394(01)01150-3

[8] Bowling BS Damato BE Foy PM Choroidal Melanoma Metastatic to the Contralateral Orbit: Implications for Patient Management Eye 1994 8 144 145 8013710 10.1038/eye.1994.29

[9] Shields JA Perez N Shields CL Singh AD Eagle RC Jr Orbital Melanoma Metastatic from Contralateral Choroid: Management by Complete Surgical Resection Ophthal Surg Lasers 2002 33 5 416 420 12358296

[10] Wachtlin J Bechrakis NE Schueler AO Helbig H Bornfeld N Foerster MH Phacoemulsification following treatment of choroidal melanoma Graefe’s Arch Clin Exp Ophthalmol 2000 238 942 948 10.1007/s004170000208 11196355

[11] Collaborative Ocular Melanoma Study Group Incidence of cataract and outcomes of cataract surgery in the first 5 years after iodine 125 brachytherapy in the collaborative ocular melanoma study Ophthalmology 2007 114 7 1363 1371 17337065 10.1016/j.ophtha.2006.10.039

